# Toxicity Evaluation of Long-Term Topical Application of Recombinant Human Keratinocyte Growth Factor-2 Eye Drops on *Macaca Fascicularis*

**DOI:** 10.3389/fphar.2021.740726

**Published:** 2021-09-21

**Authors:** Le Li, Lijia Li, Qi Chen, Xuanxin Yang, Qi Hui, Hamdi AL-Azzani, Yadong Huang, Jianqiu Cai, Xiaojie Wang, Zi Jin

**Affiliations:** ^1^ Department of Ophthalmology, the Second Affiliated Hospital and Yuying Children’s Hospital of Wenzhou Medical University, Wenzhou, China; ^2^ School of Pharmaceutical Sciences of Wenzhou Medical University, Wenzhou, China; ^3^ School of Ophthalmology and Optometry, Wenzhou Medical University, Wenzhou, China; ^4^ College of Pharmacy and Guangdong Provincial Key Laboratory of Bioengineering Medicine, Jinan University, Guangzhou, China

**Keywords:** rhKGF-2 eye drops, corneal epithelial injury, toxicity, M fascicularis, the safe dose range

## Abstract

Recombinant human keratinocyte growth factor-2 (rhKGF-2), an effective agent for the regeneration of epithelial tissue, was found to have great potential for use in treatments of corneal diseases that involve corneal epithelial defects. Furthermore, the safety of long-term and high-dose external use of KGF-2 eye drops in rabbits has been well established previously. The aim of this study is to determine the safe dose range and target organs for toxicity of rhKGF-2 eye drops in *Macaca fascicularis* (*M. fascicularis*). The *M. fascicularis* animals were administered with different doses of rhKGF-2 eye drops (125, 500, and 2000 μg/ml) for four consecutive weeks, followed by a 2 week recovery period. No significant differences in weight, electrocardiogram characteristics, blood and urine indexes, pathology, and bone marrow cells were detected among the animals in different groups. The corneas of some animals in the middle- and high-dose groups showed fluorescence when stained with sodium fluorescein, and then the staining disappeared on days 28 and 42. Anti-rhKGF-2 antibodies were detected in a small number of animals in the high-dose group, and their level decreased after rhKGF-2 withdrawal. No neutralizing antibodies were detected. The result demonstrated that there was no obvious adverse reaction when topical application of rhKGF-2 eye drops at the dosage of 125 or 500 μg/ml on the *M. fascicularis.* This study is of great significance for the future clinical transformation of rhKGF-2 eye drops.

## Introduction

Corneal epithelial injury is a frequently occurring condition that is commonly encountered in the ophthalmic clinic. If not treated promptly and effectively, this condition may lead to corneal ulcer or even corneal perforation, which severely affects the prognosis of visual function in patients (Ljubimov and Saghizadeh, 2015; Ziaei et al., 2018). In recent years, significant progress has been made in the field of corneal epithelial injury treatment. However, new drugs are still needed.

Keratinocyte growth factor-2 (KGF-2), also known as fibroblast growth factor 10 (FGF-10), is a member of the FGF family that is involved in the repair of corneal injury (Wang et al., 2010; Zheng et al., 2015). It can accelerate the proliferation and migration of epithelial cells without stimulating the overgrowth of myofibroblasts (Rajan et al., 2004; Carrington and Boulton, 2005). As an effective agent for the regeneration of epithelial tissue, KGF-2 has great potential for use in treatments of corneal diseases that involve corneal epithelial defects (Wang et al., 2010).

In a previous study, we had examined the *in vivo* effect of topically applied KGF-2 on the wound healing of injured rabbit corneal epithelium using a carbon dioxide laser. The results had shown that KGF-2 can regulate corneal epithelial wound healing by reducing ocular inflammation, stromal edema, and fibrosis, as well as by inhibiting corneal neovascularization (Wang et al., 2010). Based on these results, we had developed the first effective recombinant human KGF-2 (rhKGF-2)-based eye drop product for the topical treatment of corneal epithelial injury (Wu et al., 2009). This product is now in the process of being approved by the China Food and Drug Administration. The safety of long-term, high-dose external use of KGF-2 eye drops in rabbits has been well established previously (Cai et al., 2019). However, the KGF-2 genes in rabbits are only 90.5% similar to those in humans. Non-human primates that exhibit greater genetic similarity with humans are considered a more ideal model for evaluating the toxicity of drugs. Therefore, in this study, we use the *M. fascicularis* animal model to assess the safe dose range of rhKGF-2 eye drops and to evaluate any possible damage to the eyes and to other target organs induced by long-term topical rhKGF-2 overdose. The results reported herein are of great significance for the future clinical development of rhKGF-2.

## Methods

### Experimental Drug

The rhKGF-2 eye drops were provided by the College of Pharmacy of Wenzhou Medical University (Production Batch No. J20110201; Wenzhou, China), and they were used to prepare three rhKGF-2 solutions of varying concentrations (125, 500, and 2000 μg/ml). The solutions were preserved at 2–8°C, and the eye drop matrix containing 0 μg/ml rhKGF-2 was used as the control.

### Animals and Experimental Groups

*M. fascicularis* animals were purchased from Suzhou Xishan Zhongke Experimental Animal Co., Ltd., and they were subjected to 14 days of animal quarantine and 91 days of environmental adaptation before experimentation. The experiments were approved by the Institutional Animal Care and Use Committee of Suzhou Xishan Zhongke Pharmaceutical Research and Development Co., Ltd (Suzhou, China). IRB approval number was SAC100116 (2011–01–18).

Twenty four *M. fascicularis* were randomly divided into four groups with six animals (three males and three females) in each group, according to sex and weight stratification. The details of grouping and administered rhKGF-2 dosage in each group are shown in Table 1. KGF-2 eye drops (0.1 ml) were applied on one eye in each animal, six times a day for four consecutive weeks. After a 2 week recovery period, the animals were intravenously injected with an overdose of pentobarbital sodium (Shanghai Westang Bio-Tech Co., Ltd., Shanghai, China) for euthanasia.

**TABLE 1:** Grouping and dosage of rhKGF-2 in the long-term toxicity test.

Items	Control		rhKGF-2 groups		
			Low	Medium	High
Concentration of rhKGF-2 (μg/ml)		0	125	500	2000
Anatomical time (day)	28	♀1,201 ♀1,202	♀2,207 ♀2,208	♀3,213 ♀3,214	♀4,219 ♀4,220
		♂1,104 ♂1,105	♂2,110 ♂2,111	♂3,116 ♂3,117	♂4,122 ♂4,123
		42	♀1,203 ♂1,106	♀2,209 ♂2,112	♀3,215 ♂3,118	♀4,221 ♂4,124

### General Observation and Measurements

The mental state and activity of the animals were observed twice a day. Their weight and temperature were measured weekly using a TCS-30D-600 electronic weighing device and an Omron digital thermometer, respectively. Ocular secretion, conjunctival congestion, and other irritative reactions were visually observed before and after each administration. Electrocardiography was performed on all experimental animals at 0, 1, 2, and 4 weeks after the first administration and at the end of the recovery period (6 weeks).

### Blood and Urine Examinations

Blood and urine samples collected from the animals at 0, 1, 2, and 4 weeks after the first administration and at the end of the recovery period (6 weeks) were examined. The blood samples (2 ml) were taken from the forelimb vein, and the indicators of hematology, blood biochemistry, and blood coagulation function were analyzed using an automated biochemical analyzer (Abbott ARCHITECT c8000, Abbott Core Laboratory, Chicago, United States). During the process of animal urine collection, drinking water was cut off. Fresh and clean urine samples were analyzed using the Aution MAX AX-4280 Urine analyzer (ARKRAY Shanghai Co., Ltd., Shanghai, China).

### Ophthalmic Examinations

Ophthalmic examinations of the anterior and posterior segments were performed at 0, 1, 2, 3, and 4 weeks after the first administration and at the end of the recovery period (6 weeks). Anterior segment examination included observations of the conjunctiva, eyelid, cornea, sclera, iris, anterior chamber, and lens using a S350S slit-lamp microscope (MediWorks Co., Ltd., Shanghai, China), whereas posterior segment examination included observations of the vitreous body and fundus (optic disc, macula, and retinal blood vessel) using a YZ11 funduscope (Liuliu Vision Technology Co., Ltd., Suzhou, China).

### Pathological Examination

All of the animals in each group were euthanized and dissected at the end of the administration and recovery periods. Before dissection, the appearance of the animals was evaluated. After dissection, the subcutaneous tissues of the thorax and abdomen were observed, and the chest, abdomen, pelvic, and cranial cavities were opened to examine the internal organs. The epididymis, ovaries, adrenals, thymus, uterus, testicles, heart, liver, spleen, lung, kidney, and brain were weighed, and the ratios of the weight of each organ to the weight of the brain were calculated. The above-mentioned organs and tissues, as well as the cornea, palpebral conjunctiva, nasal mucosa, and nasopharyngeal tissue, were all excised. First, they were fixed in 10% formalin, dehydrated, and embedded in paraffin. Then, they were cut into 5 μm slices for hematoxylin and eosin staining, followed by histopathological examination.

### Bone Marrow Examination

The sternum was removed from each animal during dissection, and a bone marrow smear was prepared using the Nikon ECLIPSE Ti microscope (Nikon, Tokyo, Japan). The numbers of plasmocytes, lymphocytes, erythrocytes, and neutrophils at the end of the rhKGF-2 administration and recovery periods were determined.

### Immunogenicity and Neutralizing Antibody Test

At 0, 1, 2, 3, and 4 weeks after the first administration and at the end of the 2 week recovery period, serum samples of the *M. fascicularis* animals were collected, and immunogenicity tests were conducted by enzyme-linked immunoassay. The envelope antigen of rhKGF-2 (concentration of 8 μg/ml) was deposited into 96-well plates (Thermo Fisher Scientific, Waltham, MA, United States) with 100 μL per well. The antigen was incubated overnight at 4°C (6 parallel wells), and after being blocked with 1.0% bovine serum albumin (BSA), 100 μL of serum samples (diluted 10, 20, 40, 80, 160, 320, and 640 times) were added to each well. After incubation at 37°C for 1 h, the reaction liquid was discarded, and the plate was washed three times with 0.05% phosphate-buffered saline containing Tween detergent. Subsequently, the plate was incubated with 100 μL of goat anti-monkey IgG-horseradish peroxidase antibody (1:2000; Abcam, Cambridge, MA, United States), which was diluted with 0.1% BSA, at 37°C for 1 h. Tetramethylbenzidine and 2 M H_2_SO_4_ were used for color reaction and termination, respectively. Dual-wavelength colorimetric absorbance measurement was conducted at 450 and 630 nm using a Bio-Tek ELx 800-type microplate reader (Bio-Rad, Hercules, CA, United States). The neutralizing antibody of rhKGF-2 in blood samples was measured by the Thiazolyl Blue Tetrazolium Bromide (MTT) assay, using BaF3 cells transfected with FGFR2IIIb (cell source) This antibody was considered present when the proliferation activity of BaF3 cells was below 50% compared to the control group.

### Statistical Methods

All of the data were statistically analyzed using SPSS software (version 13.0; SPSS, Inc., Chicago, IL, United States). One-way analysis of variance was used to evaluate the differences in variables among the four groups and at different time points of measurement. Homogeneity was tested using Levene’s variance homogeneity test, and when the variance was uniform, the variance analysis results were directly quoted. If the case of non-uniform variance, the overall difference was determined according to Welch’s test results. The Bonferroni test was further used to compare the differences between the groups and time points. *p* < 0.05 denotes statistical significance (*), and *p* < 0.01 denotes high significance (**).

## Results

### General Clinical Signs and Measurements

**FIGURE 1:**
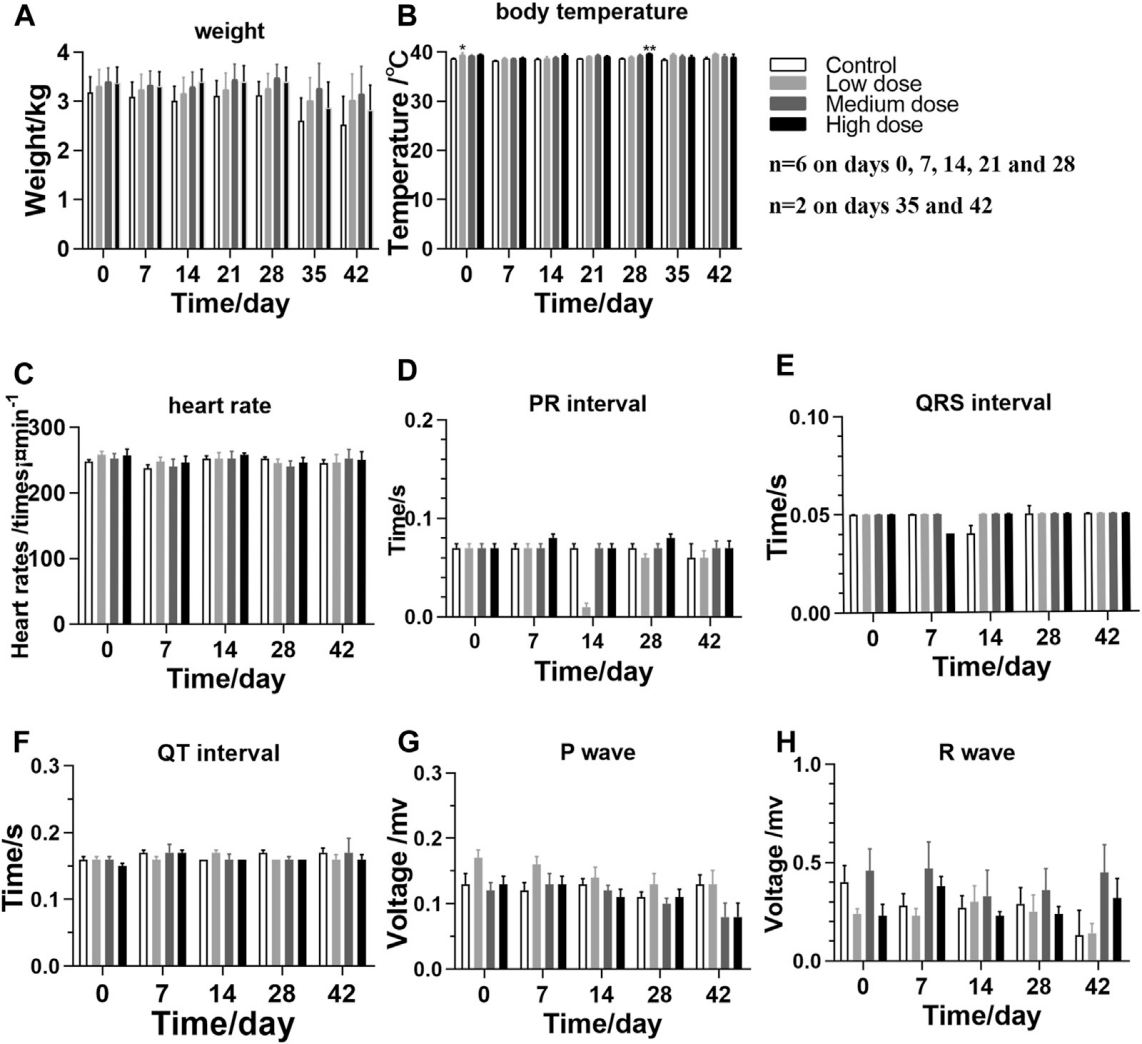
General clinical signs and measurements of *M. fascicularis* throughout the long-term toxicity test **(A)** weight **(B)** body temperature **(C)** heart rate, and **(D**–**H)** ECG. *p < 0.05 and **p < 0.01, compared to the control group.

The animals in different groups showed normal behavior, activity, and diet after rhKGF-2 administration, and they were in a good mental state. Occasionally, loose stools occurred before and after administration. Each animal consumed 200 g of food daily, and the weights of all rhKGF-2-treated animals were similar to those of the animals in the control group (Figure 1A; *p* > 0.05). Before the first administration, the body temperature of the medium-dose group was higher than that of the control group (Figure 1B; *p* < 0.05). And the high-dose group showed higher body temperature, compared to the control group, at the end of administration (Figure 1B; *p* < 0.01).

Based on the recorded electrocardiograms, the levels of indicators in the rhKGF-2-treated groups are not significantly different than those detected in the control group (Figures 1C–H; *p* > 0.05), which indicates that the eye drops have no toxic effect on the heart.

### Indicators in the Blood and Urine

The levels of blood biochemical indicators including alanine aminotransferase (ALT), aspartate aminotransferase (AST), lactate dehydrogenase (LDH), and creatine kinase (CK) detected in different groups are listed in Table 2. Basically, the blood biochemical and hematological parameters, as well as the coagulation function, in the rhKGF-2 treatment groups are similar to those in the control group (Table 2 and Supplementary Tables S1, S2, S3, S4, and S5; *p* > 0.05). In addition, the urine test results show no significant abnormalities in the rhKGF-2-treated groups compared to the control group (Supplementary Tables S6 and S7).

**TABLE 2:** Blood biochemical index of *Macaca fascicularis*. Specific blood biochemical index, including ALT, AST, LDH and CK, of *Macaca fascicularis* on days 0, 7, 14, 28, and 42 in long-term toxicity test (n = 6 before day 28, n = 2 on day 42).

Index	Time/d	Group			
		Control	Low dose	Medium dose	High dose
ALT U/L	0	51 ± 33	37 ± 12	45 ± 15	74 ± 63
	7	89 ± 33	83 ± 9	79 ± 19	86 ± 21
	14	70 ± 45	51 ± 16	60 ± 25	72 ± 45
	28	62 ± 40	32 ± 8	46 ± 22	71 ± 93
	42	89	41	34	43
AST U/L	0	37 ± 14	31 ± 5	39 ± 7	39 ± 9
	7	66 ± 26	63 ± 21	67 ± 16	64 ± 15
	14	45 ± 12	53 ± 28	45 ± 11	42 ± 10
	28	47 ± 16	32 ± 5	41 ± 13	40 ± 18
	42	40	36	28	26
LDH U/L	0	461 ± 186	415 ± 173	405 ± 88	404 ± 118
	7	1,144 ± 739	977 ± 428	716 ± 226	710 ± 116
	14	860 ± 936	750 ± 376	589 ± 271	480 ± 137
	28	953 ± 983	513 ± 135	482 ± 179	391 ± 73
	42	428	297	303	223
CK U/L	0	172 ± 77	158 ± 48	206 ± 98	393 ± 521
	7	275 ± 128	535 ± 404	317 ± 150	495 ± 210
	14	242 ± 135	364 ± 307	215 ± 90	202 ± 102
	28	213 ± 245	111 ± 31	129 ± 19	162 ± 62
	42	142	142	178	289

### Ophthalmic Examinations

**FIGURE 2:**
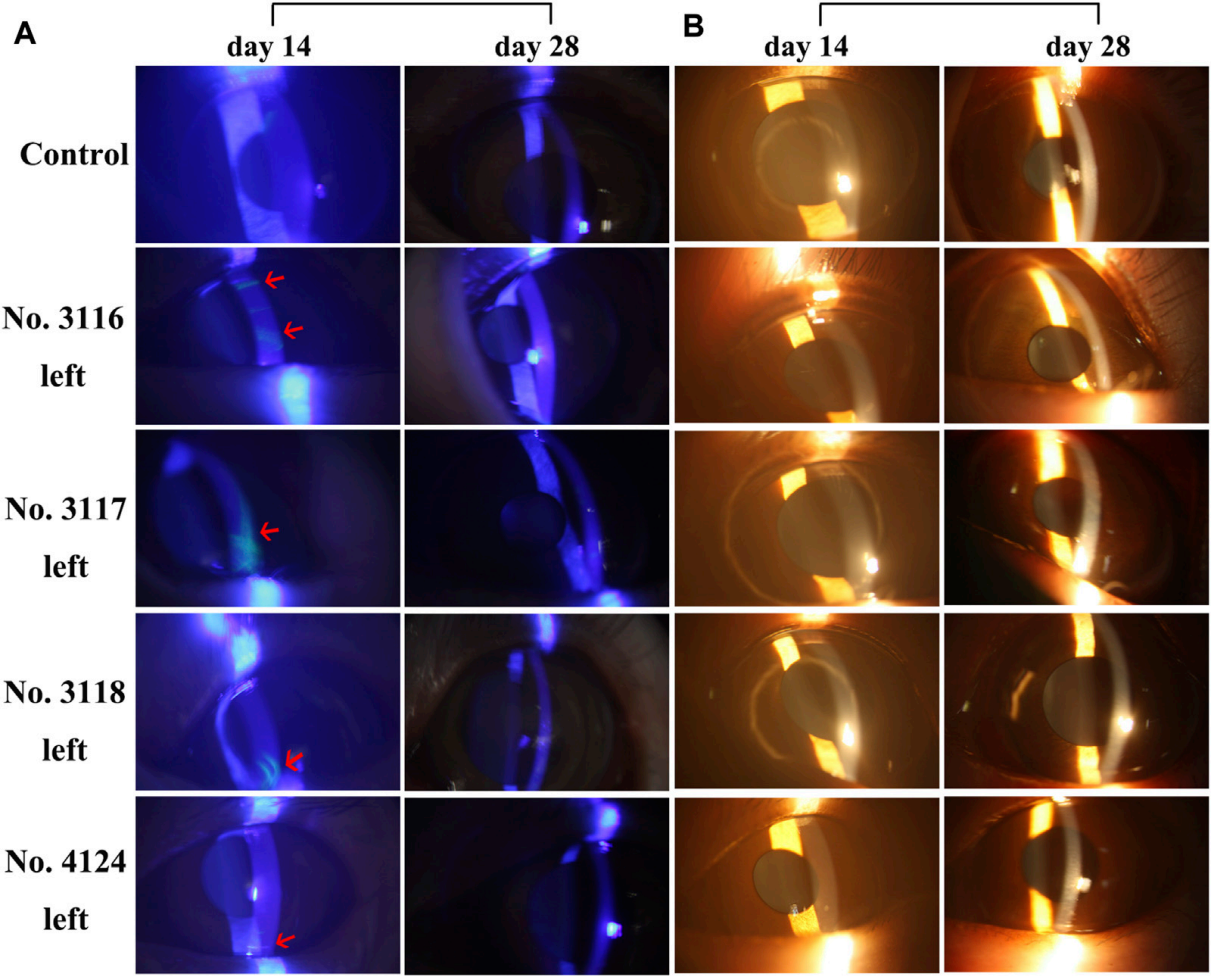
Slit-lamp microscope images of corneas of **5** *M. fascicularis* on days 14 and 28 **(A)** Fluorescein sodium staining images of the corneas. 2% sodium fluorescein solution was dropped into the conjunctival sac, followed by observation under the cobalt blue light of a slit lamp microscope. Three animals (no. 3116, 3,117, and 3,118) in the middle-dose group and one animal (no. 4124) in the high-dose group showed fluorescence on day 14. Whereas, the staining disappeared on day 28. The arrow indicates the area stained by fluorescein sodium **(B)** Corneal images under the yellow light of slit-lamp. There was no difference in corneal clarity between the control group and the three rhKGF-2 treatment groups.

To determine whether long-term use of rhKGF-2 would cause damage to the cornea, 2% fluorescein sodium was applied on the corneas of the animals. The defect of the corneal epithelium would show yellow-green fluorescence when irradiated with cobalt blue light under the slit lamp. As shown in Figure 2A, the local sheet fluorescein sodium staining was been observed on the left corneas of three animals in the medium-dose group and one animal in the high-dose group under blue light on day 14. However, the fluorescein sodium staining disappeared in all four animals on day 28, indicating that the corneal injury had been repaired. In addition, when examined and photographed with yellow light under the slit-lamp, it was found that there was no difference in corneal clarity between the control group and the three administration groups (Figure 2B). The eyelid, conjunctiva, cornea, sclera, iris, anterior chamber, and lens were found to be in normal condition throughout the experiment, as were the vitreous body, optic disc, macula, and retinal vessels observed using a YZ11 funduscope after mydriasis with tropicamide.

### Pathological Examination

None of the animals died during the experiment, and at the end of the administration and recovery periods, all 24 *M. fascicularis* were sacrificed for examination. Macroscopic observation of the tissues on days 28 and 42 shows that none of the animals exhibits obvious pathological changes such as necrosis, adhesion, mass formation, organ loss, and dysplasia.

**FIGURE 3:**
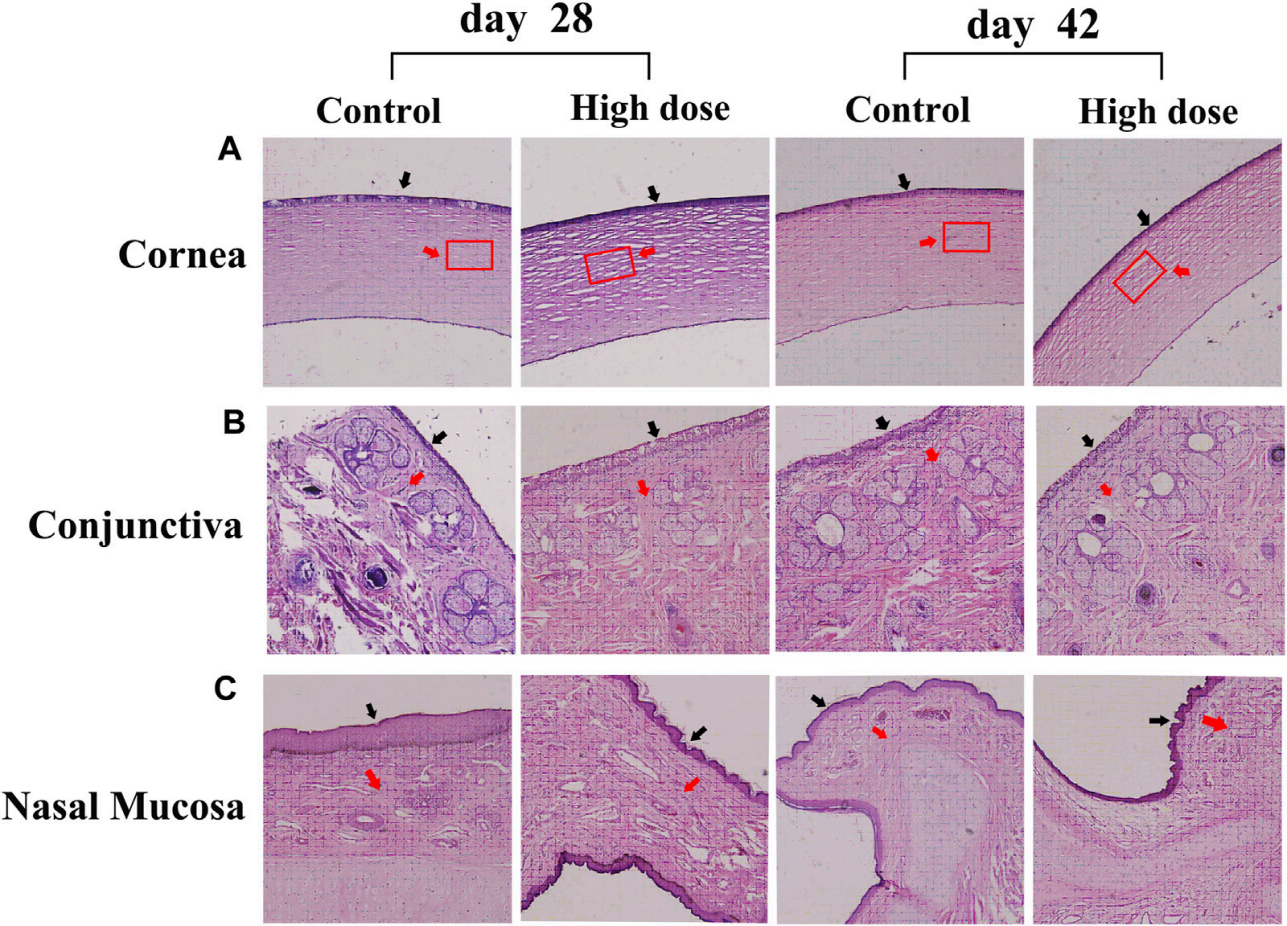
Hematoxylin-eosin staining of the cornea, conjunctiva, and nasal mucosa in the control and high-dose groups on days 28 and 42 **(A)** No obvious defects and overproliferation of corneal epithelial cells were detected on days 28 and 42, but the stromal layer of the high-dose group was looser than that of the control group. The black arrow indicates the epithelial layer. The red arrow indicates the matrix layer. The rectangle represents the area where the degree of compactness changes in the matrix layer **(B)** Conjunctiva. Animals in the high-dose group exhibit normal morphology of conjunctiva on days 28 and 42. The black arrow indicates the epithelial layer. The red arrow indicates lamina propria **(C)** Nasal mucosa. Animals in the high-dose group exhibit normal morphology of conjunctiva on days 28 and 42. The black arrow indicates the epithelial layer. The red arrow indicates lamina propria. Magnification: ×100.

Pathological examination of the corneal sections shows no obvious defects and overproliferation of corneal epithelial cells on days 28 and 42, but the stromal layer of the high-dose group was looser than that of the control group (Figure 3A).

**FIGURE 4:**
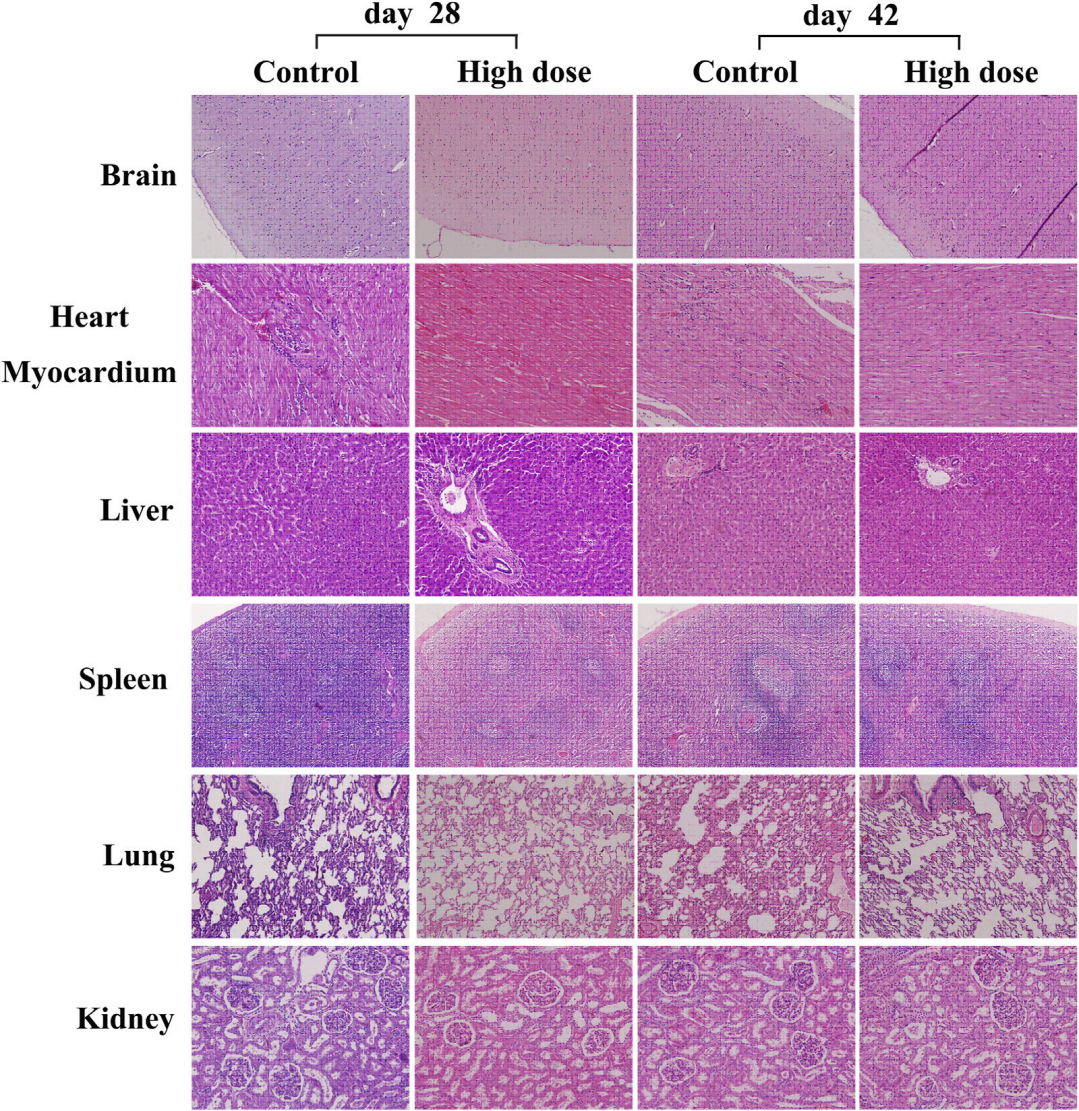
Hematoxylin-eosin staining of some vital organs in *M. fascicularis*, including brain (100x), heart myocardium (200x), liver (200x), spleen (100x), lung (100x), and kidney (200x). No obvious inflammatory cell infiltration.

**FIGURE 5:**
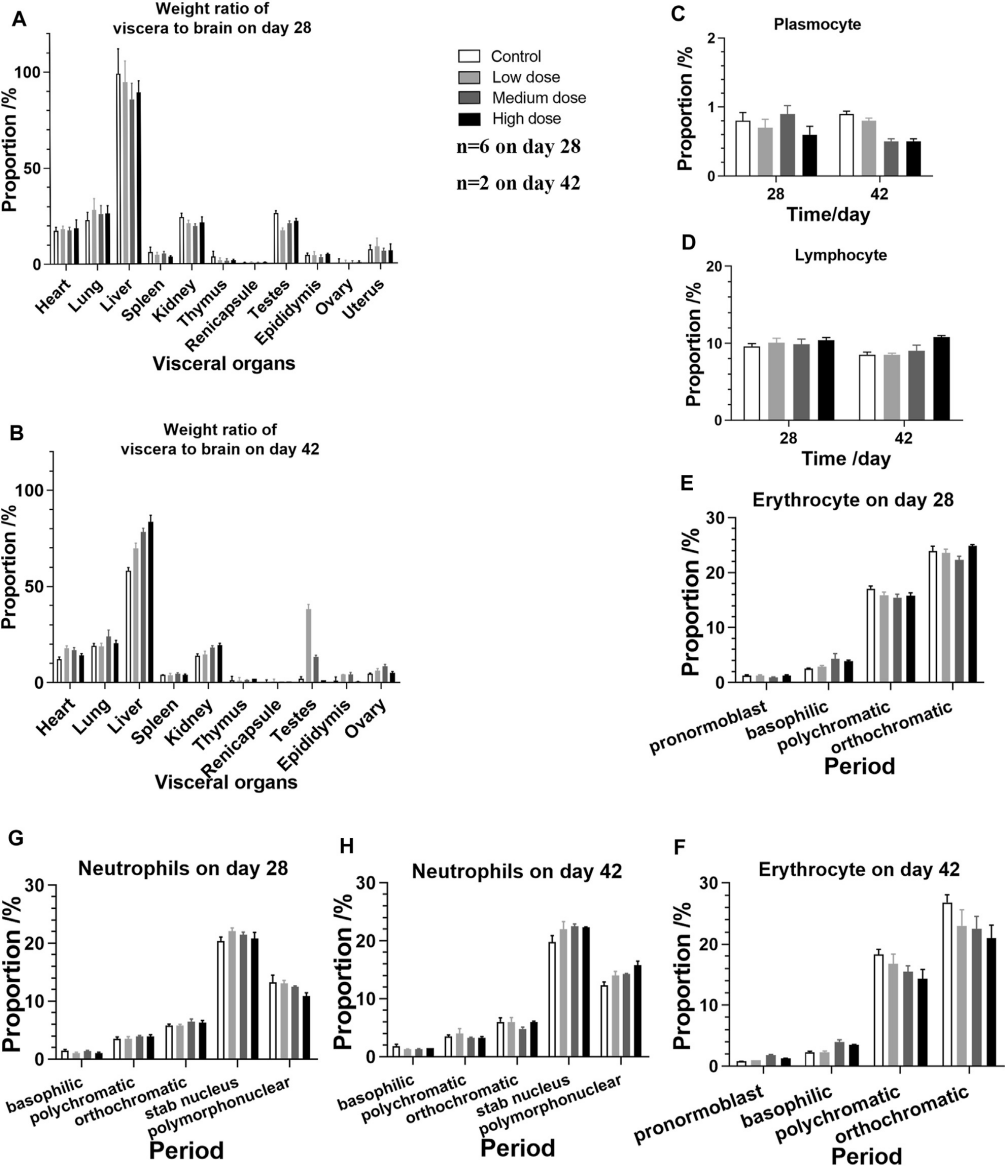
Viscera-brain ratios and bone marrow cell test performed on animals in the four groups on days 28 and 42 **(A)** Viscera-brain ratios determined after 28 days of consecutive administration (n = 4 on day 28, n = 2 of gonad) **(B)** Viscera-brain ratios determined on day 42, after the administration is stopped (n = 2 on day 28, n = 1 of gonad) **(C–H)** Bone marrow cells (n = 6 before day 28, n = 2 on day 42) **(C)** plasmocytes **(D)** lymphocytes **(E)** erythrocytes on day 28 **(F)** erythrocytes on day 42 **(G)** neutrophils on day 28, and **(H)** neutrophils on day 42.

The analysis of visceral organ tissue sections demonstrates that there are no significant differences in the pathological changes of all tissues (Figure 4 and Supplementary Figure S1), as shown in Tables 3, 4 that list the individual spontaneous pathological changes observed on days 28 and 42. Furthermore, the viscera to brain weight ratios determined for animals in different rhKGF-2 treatment groups on days 28 and 42 are significantly different than those determined for animals in the control group (Figures 5A,B), which indicates that the rhKGF-2 eye drops have little effect on the weight of visceral organs.

**TABLE 3:** Pathological changes in each organ after 28 consecutive days of administration in the *Macaca fascicularis* long-term toxicity test.

Organs (positions) pathologic	Number		Control		Low dose		Medium dose		High dose	
	♀(8)	♂(8)	♀(2)	♂(2)	♀(2)	♂(2)	♀(2)	♂(2)	♀(2)	♂(2)
Heart (apex, ventricular, septal)	–	–	–	–	–	–	–	–	–	–
Inflammatory cells	8	8	2	1	1	0	1	0	0	1
Lung (left, right)	–	–	–	–	–	–	–	–	–	–
Inflammatory cells and alveolar interval	8	8	0	0	0	0	0	1	0	0
Bleeding or edema	8	8	1	1	0	2	0	1	1	1
Liver (left, right)	–	–	–	–	–	–	–	–	–	–
Granuloma	8	8	0	0	0	0	1	0	0	0
Kidney (left, right)	–	–	–	–	–	–	–	–	–	–
Chronic inflammatory cells or organize shrinking	8	8	1	1	1	0	2	1	1	0
Epithelial cells of renal tubule drop off	8	8	0	0	0	0	2	0	0	0
Prostate	–	–	–	–	–	–	–	–	–	–
Chronic inflammatory cells	0	8	0	0	0	0	0	1	0	1
Cervix	–	–	–	–	–	–	–	–	–	–
Acute chronic inflammatory cells or fester	8	0	2	0	1	0	1	0	0	0
Thymus	–	–	–	–	–	–	–	–	–	–
Tissue degradation	8	8	0	2	1	1	1	1	1	0
Adenoid structure formation	8	8	0	2	1	1	1	2	1	0
Thyroid gland	–	–	–	–	–	–	–	–	–	–
Inflammatory cells	8	8	0	0	1	1	0	1	0	1
Salivary gland	–	–	–	–	–	–	–	–	–	–
Chronic inflammatory cell or acinic destroy	8	8	1	2	0	0	1	2	1	0
Bladder	–	–	–	–	–	–	–	–	–	–
Chronic inflammatory cell	8	8	0	0	1	0	0	0	0	1
Esophageal	–	–	–	–	–	–	–	–	–	–
Chronic inflammatory cell	8	8	0	0	1	0	0	0	0	1

**TABLE 4:** Pathological changes in each organ on day 42 in the recovery period of the *Macaca fascicularis* long-term toxicity test.

Organs (positions) pathology	Number		Control		Low dose		Medium dose		High dose	
	♀(4)	♂(4)	♀(1)	♂(1)	♀(1)	♂(1)	♀(1)	♂(1)	♀(1)	♂(1)
Heart (apex, ventricular, septal)	–	–	–	–	–	–	–	–	–	–
Inflammatory cells	4	4	1	1	0	1	0	0	0	0
Lung (left, right)	–	–	–	–	–	–	–	–	–	–
Inflammatory cells and alveolar interval	4	4	0	0	1	0	0	0	0	0
Bleeding or edema	4	4	0	1	0	1	0	1	0	1
Liver (left, right)	–	–	–	–	–	–	–	–	–	–
Granuloma	4	4	0	0	1	0	0	0	0	0
Kidney (left, right)	–	–	–	–	–	–	–	–	–	–
Chronic inflammatory cells or organize shrinking	4	4	0	0	0	1	1	0	0	0
Prostate	–	–	–	–	–	–	–	–	–	–
Chronic inflammatory cells	0	4	0	0	0	0	0	1	0	0
Glands immature and small	0	4	0	1	0	0	0	0	0	1
Testes	0	4	0	1	0	0	0	0	0	1
Spermatogenic tubule immature and testicular volume small										
Epididymis	0	4	0	1	0	0	0	0	0	1
No sperm										
Cervix	–	–	–	–	–	–	–	–	–	–
Acute chronic inflammatory cells or fester	4	0	1	0	1	0	1	0	1	0
Thymus	–	–	–	–	–	–	–	–	–	–
Tissue degradation	4	4	0	0	0	0	1	0	0	0
Adenoid structure formation	4	4	1	1	0	1	1	0	0	0
Thyroid gland	–	–	–	–	–	–	–	–	–	–
Inflammatory cells	4	4	0	1	0	0	0	0	0	0
Salivary gland	–	–	–	–	–	–	–	–	–	–
Chronic inflammatory cell or acinic destroy	4	4	1	0	0	1	1	1	1	1

Considering that the eye drops may flow through the nasolacrimal duct, the morphology of conjunctiva and nasal mucosa of *M. fascicularis* was observed. The obtained results show that all animals in the high-dose group exhibit normal morphology of conjunctiva and nasal mucosa on days 28 and 42, similar to the animals in the control group (Figures 3B,C). This suggests that the rhKGF-2 eye drops do not injure the conjunctiva and nasal mucosa.

### Determination of the rhKGF-2 Antibody in the Blood

Anti-rhKGF-2 antibody detection in the blood is an important immunological index that reflects long-term toxicity. On day 28 after administration, the maximum dilutions of animals no. 4122 and 4,124 in the high-dose group were 1:160 and 1:80, respectively. However, the antibody of the titer in the animal 4,124 decreased during the recovery period (Table 5). Interestingly, no neutralizing antibodies were detected (data not shown), which might help maintain the activity of the rhKGF-2 protein in the eye drops.

**TABLE 5:** The anti-rhKGF-2 antibody in the blood of the *Macaca fascicularis* on days 14, 21, 28 and 42. - negative; NT, not detected.

Concentration of rhKGF-2 (μg/ml)	Animal no.	Anti-rhKGF-2 antibody maximum titer			
		Day 14	Day 21	Day 28	Day 42
0	1,201	–	–	–	NT
	1,202	–	–	–	NT
	1,203	–	–	–	–
	1,104	–	–	–	NT
	1,105	–	–	–	NT
	1,106	–	–	–	–
125	2,207	–	–	–	NT
	2,208	–	–	–	NT
	2,209	–	–	–	–
	2,110	–	–	–	NT
	2,111	–	–	–	NT
	2,112	–	–	–	–
500	3,213	–	–	–	NT
	3,214	–	–	–	NT
	3,215	–	–	–	–
	3,116	–	–	–	NT
	3,117	–	–	–	NT
	3,118	–	–	–	–
2000	4,219	–	–	–	NT
	4,220	–	–	–	NT
	4,221	–	–	–	–
	4,122	–	–	1:160	NT
	4,123	–	–	–	NT
	4,124	–	–	1:80	1:40

### Bone Marrow Examination

Bone marrow cell examination was performed, and no significant myeloproliferation was observed. Moreover, the numbers of plasmocytes, lymphocytes, erythrocytes, and neutrophils detected in the rhKGF-2 treatment groups are not statistically different than those measured in the control group (Figures 5C–H; *p* > 0.05).

## Discussion

Long-term toxicity study is an essential component of non-clinical safety evaluation of rhKGF-2 eye drops. In general, the results of a long-term toxicity study can be used to predict the initial dose and safe dose range of rhKGF-2 eye drops in clinical trials.

As a corneal injury treatment, the rhKGF-2 eye drops are generally applied 4 to 6 times a day for one or 2 weeks. In this long-term toxicity study, the administration cycle of rhKGF-2 eye drops was set for 1 month. Based on a previous pharmacodynamics study, the best effective concentration of rhKGF-2 for corneal wound repair is 25 μg/ml (Wang et al., 2010). The low, medium and high doses of rhKGF-2 tested herein are respectively 5 (125 μg/ml), 20 (500 μg/ml), and 80 (2000 μg/ml) times as high as the pharmacodynamic equivalent dosage.

In this study, the safety of the rhKGF-2 eye drops was mainly evaluated based on general clinical observation, as well as hematuria, ophthalmology, pathology, and immunogenicity examinations. Throughout the experiment, no significant differences in mental state, weight, food intake, and ECG were noted among the animals in different groups. However, the body temperature of the high-dose group was found to be greater than that of the control group at the end of the administration period, possibly due to individual differences between animals.

To determine whether long-term use of rhKGF-2 contributes to corneal damage, 2% fluorescein sodium was applied on the corneas of animals. On day 14, the corneas of three animals (no. 3116, 3,117, and 3,118) in the middle-dose group and one animal (no. 4124) in the high-dose group showed fluorescence, which indicates that the corneal epithelium of these animals was damaged. However, the staining disappeared at the end of the administration and recovery periods. Although a few animals showed corneal damage, the results suggest that such damage is independent of rhKGF-2 dosage and exposure time. In addition, the fluorescein staining eventually disappeared, thus we further analyzed the data of immunogenicity and histopathology examinations to determine the safe dose range of rhKGF-2.

Immunogenicity testing plays an essential role in verifying the non-clinical safety of therapeutic protein drugs, and it is used to determine the incidence, titer, duration, and neutralization ability of anti-drug antibodies (Guinn et al., 2021). In this study, the anti-rhKGF-2 antibodies were only detected in animals no. 4122 and 4,124 in the high-dose group on day 28, and the antibodies in animal no. 4124 still could be detected at the end of the recovery period. Therefore, we speculate that the corneal staining may be related to immune response. However, no neutralizing antibodies were detected throughout the experiment, which suggests that the effectiveness of the drug is not compromised. In future clinical trials, the aspect of immunogenicity must be considered more attentively.

The receptor of KGF-2 (FGFR2 III b) is mainly expressed in epithelial tissue (Itoh, 2016). Therefore, the safety of the KGF-2 on epithelial overproliferation must be considered. HE staining and slit-lamp microscopic observations revealed that KGF-2 could not induce epithelial overproliferation of the cornea, conjunctiva, or nasal mucosa in *M. fascicularis*.

Considering the various roles of rhKGF-2 in multiple organs including the lung (Ramasamy et al., 2007; El Agha et al., 2014; Yuan et al., 2018), limbs (Sekine et al., 1999; Norton et al., 2005), heart (Marguerie et al., 2006; Rochais et al., 2014), liver (Berg et al., 2007; Yang et al., 2021), lacrimal gland, and salivary gland (Garg et al., 2017; Seymen et al., 2017), tissue slices of these organs were stained with HE to observe any morphologic changes induced by long-term rhKGF-2 administration. The obtained results demonstrate that rhKGF-2 exhibits no toxicity in *M. fascicularis.* In the previous study, the highest content of rhKGF-2 was found in the cornea, and little entered the body through blood circulation (Cai et al., 2015). Therefore, rhKGF-2 may have fewer influences on systemic organs when used for eye drops compared to administered by injection.

In conclusion, the results reported herein suggest that there was no obvious adverse reaction when topical application of rhKGF-2 eye drops at the dosage of 125 or 500 μg/ml on the *M. fascicularis*. Increasing the dosage to 2000 μg/ml may induce the production of anti-rhKGF-2 antibodies. This study is of great significance for the future clinical transformation of rhKGF-2 eye drops.

## Data Availability

The original contributions presented in the study are included in the article/Supplementary Material, further inquiries can be directed to the corresponding authors.
