# Supplementary material for: Toxicity Evaluation of Long-Term Topical Application of Recombinant Human Keratinocyte Growth Factor-2 Eye Drops on Macaca Fascicularis
Source: Front Pharmacol. 2021 Sep 21;12:740726. doi: 10.3389/fphar.2021.740726 (PMC8490875; doi:10.3389/fphar.2021.740726)
Supplement: Supplementary file 1 [file DataSheet1.PDF]

Supplementary Materials

Fig S1. Hematoxylin–eosin staining of sexual organs in *M. fascicularis*, including uterus (40x), cervix (100x), ovary (40x), testes (100x), epididymis (100x), and prostate (100x).

Table S1. Hematology results part 1. Hematology indexes of basophils (BASO), eosinophils (EO), red blood cell specific volume (HCT), hemoglobin (HGB), lymphocytes (LYMPH), mean corpuscular hemoglobin (MCH), mean corpuscular hemoglobin concentration (MCHC), and mean corpuscular volume (MCV). $*p < 0.05$, compared with control group (mean ± SD, n = 6 before day 28; mean, n = 2 on day 42).

Table S2. Hematology results part 2. Hematology indexes of monocytes (MONO), neutrophils (NEUT), platelet count (PLT), erythrocyte count (RBC), reticulocytes (RET), and white blood cells (WBC) (mean ± SD, n = 6 before day 28; mean, n = 2 on day 42).

Table S3. Coagulation function in *M. fascicularis* after long-term toxicity testing. Prothrombin time (PT), activated partial thromboplastin time (APTT), thrombin time (TT), and fibrinogen (FIB) (mean ± SD, n = 6 before day 28; mean, n = 2 on day 42).

Table S4. Blood biochemical results part 1. Total bilirubin (TBIL), total protein (TP), albumin (ALB), globulin (GLOB), ratio of ALB to GLOB (A/G), alkaline phosphatase (ALP), glutamyl transpeptidase (GGT), and urea (mean ± SD, n = 6 before day 28; mean, n = 2 on day 42).

Table S5. Blood biochemical results part 2. Creatinine (CREA), glucose (GLU), triglyceride (TG), total cholesterol (CHOL), potassium ion (K^+), sodium (Na^+), and chloride ion (Cl^-) (mean ± SD, n = 6 before day 28; mean, n = 2 on day 42).

Table S6. Urine test results of control and low-dose group animals (day 28). Urine color, turbidity, pH, nitrite (NIT), glucose (GLU), specific gravity (SG), blood (BLD), protein (PRO), bilirubin (BIL), urobilinogen (URO), ketone (KET), and leukocyte (LEU). C, colorless; Y, yellow; LY, light yellow; N, normal; NEG, negative.

Table S7. Urine test results of medium-dose and high-dose group animals (day 28). Urine color, turbidity, pH, nitrite (NIT), glucose (GLU), specific gravity (SG), blood (BLD), protein (PRO), bilirubin (BIL), urobilinogen (URO), ketone (KET), and leukocyte (LEU). C, colorless, Y,– yellow, LY, light yellow, N, normal; NEG, negative.

Fig S1.

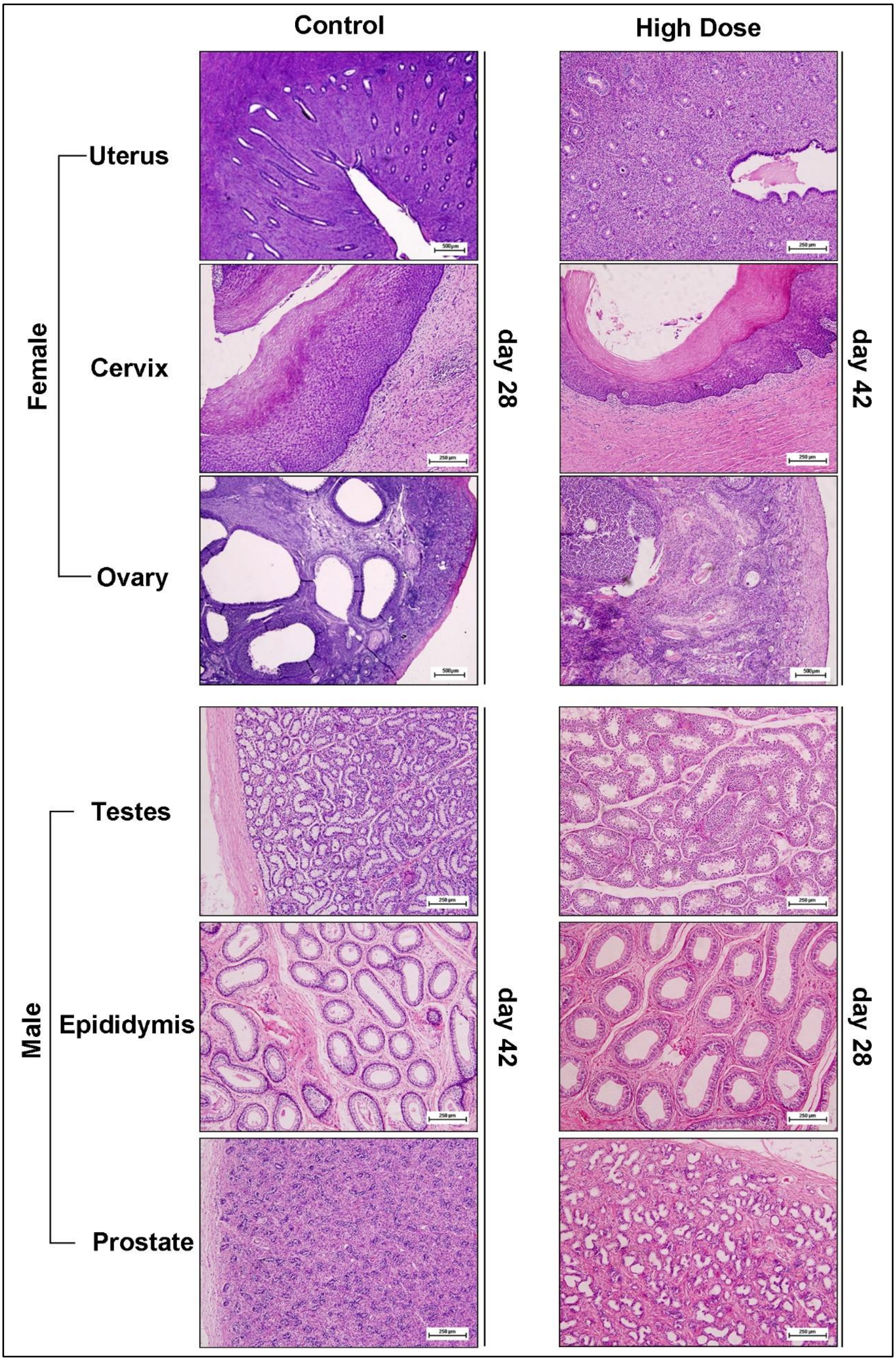
Control
High Dose
Uterus
Cervix
Ovary
Female
day 28
day 42
Testes
Epididymis
Prostate
Male
day 42
day 28

Table S1

Group	Time<br>d	BASO<br>%	EO<br>%	HCT<br>%	HGB<br>g/L	LYMPH<br>%	MCH<br>pg	MCHC<br>g/L	MCV<br>fL
Control	0	0.1±0.1	2.6±1.6	45.3±4.5	137.0±1	63.6±11.	24.0±0.7	302.5±8.	79.2±1.4
Low dose		0.1±0.0	2.6±1.9	41.9±1.7	125.0±4.	69.6±6.6	23.4±0.8	298.4±5.	78.4±3.0
Medium dose		0.1±0.1	2.6±2.3	41.4±1.7	125.5±5.	66.0±8.4	23.5±1.4	303.5±5.	77.4±4.9
High dose		0.1±0.0	1.0±0.6	43.1±1.7	131.7±7.	66.1±20.	23.0±1.1	305.3±9.	75.2±1.9
Control	7	0.2±0.1	1.8±1.2	36.9±7.3	108.2±2	53.4±10.	23.9±0.7	291.9±1	81.9±2.6
Low dose		0.1±0.1	1.8±1.4	35.2±2.6	103.4±9.	51.0±9.2	23.3±0.9	293.2±5.	79.5±2.6
Medium dose		0.1±0.1	1.7±1.5	37.4±2.4	110.5±8.	49.4±6.3	23.4±1.2	295.7±5.	79.2±4.8
High dose		0.1±0.1	0.7±0.5	38.8±2.5	115.3±9.	56.9±8.2	22.9±1.1	297.6±9.	77.1±2.1
Control	14	0.1±0.1	1.5±1.1	38.0±5.8	109.7±2	46.1±21.	23.8±1.0	286.3±1	83.0±3.1
Low dose		0.2±0.1	2.6±2.5	35.9±4.1	104.1±1	49.6±15.	22.9±1.3	289.3±4.	79.2±3.7
Medium dose		0.1±0.0	2.8±3.6	37.0±2.5	108.0±9.	41.9±12.	23.3±1.2	291.4±7.	80.1±5.4
High dose		0.1±0.1	0.8±0.7	38.1±2.5	111.9±9.	43.0±16.	22.9±1.1	293.7±9.	78.1±1.8
Control	28	0.1±0.0	1.6±0.8	40.1±4.4	117.0±17	57.5±18.	23.6±1.5	290.9±1	81.1±2.8
Low dose		0.1±0.0	2.9±2.0	38.7±3.6	110.5±12	56.0±17.	22.5±1.6	285.8±11	78.7±3.4
Medium dose		0.1±0.1	3.4±2.1	38.5±2.9	112.5±10	56.0±12.	23.1±1.2	292.6±7.	79.1±5.1
High dose		0.1±0.0	1.5±1.1	40.5±3.4	119.7±12	61.2±8.5	22.9±1.3	295.1±1	77.5±2.3
Control	42	0.2	0.8	42.0	127.5	65.7	24.0	303.5	79.1
Low dose		0.2	1.9	41.1	121.5	62.0	22.9	295.5	77.4
Medium dose		0.1	3.0	36.5	111.5	58.9	23.2	305.0	76.0
High dose		0.1	0.3	41.7	130.0	67.0	22.7	311.5	72.8

Table S2

Group	Time d	MONO %	NEUT %	PLT 10^9/L	RBC 10^{12}/L	RET 10^9/L	RET %	WBC 10^9/L
Control	0	6.3±1.0	27.5±12.4	365±48	5.71±0.48	47.3±17.1	0.84±0.33	12.45±6.9
Low dose		6.1±1.3	21.7±7.3	333±78	5.35±0.33	50.5±22.0	0.94±0.38	13.19±2.4
Medium dose		6.8±1.7	24.6±9.3	352±42	5.35±0.27	71.8±48.3	1.36±0.94	9.87±3.16
High dose		5.6±1.8	27.2±21.0	335±101	5.73±0.20	49.3±20.6	0.87±0.38	12.27±2.9
Control	7	7.3±3.3	37.4±7.3	503±129	4.51±0.96	158.1±11	4.31±4.63	10.28±3.9
Low dose		5.7±1.6	41.4±9.3	494±146	4.43±0.33	147.4±10	3.44±2.69	12.47±3.1
Medium dose		6.9±1.7	42.0±8.8	459±88	4.73±0.37	108.1±36.	2.29±0.76	9.79±2.03
High dose		4.1±1.6	38.2±9.5	425±83	5.03±0.4	125.6±55.	2.55±1.30	11.90±3.9
Control	14	7.6±2.9	44.8±20.5	469±54	4.60±0.79	148.4±11	3.72±3.67	11.77±6.6
Low dose		6.1±2.7	41.5±17.0	606±279	4.53±0.44	122.5±53.	2.72±1.24	11.97±2.2
Medium dose		5.4±1.1	49.9±16.1	499±68	4.65±0.48	116.2±43.	2.58±1.14	11.24±2.7
High dose		5.1±2.4	51.0±16.2	502±108	4.88±0.30	122.4±28.	2.52±0.59	12.42±5.2
Control	28	9.6±3.9	31.2±15.4	430±131	4.94±0.50	75.8±32.2	1.61±0.95	11.34±4.8
Low dose		6.7±1.0	34.3±18.1	407±126	4.92±0.46	84.3±50.0	1.70±0.99	12.30±2.4
Medium dose		8.8±2.7	31.8±13.8	351±71	4.88±0.49	87.9±20.3	1.83±0.52	9.50±2.13
High dose		5.8±1.3	31.5±6.5	336±87	5.23±0.47	87.7±39.7	1.66±0.67	10.30±2.5
Control	42	7.5	25.9	360	5.31	39.2	0.75	7.24
Low dose		4.1	31.9	292	5.31	44.6	0.83	12.60
Medium dose		4.7	33.4	364	4.82	41.5	0.85	8.95
High dose		4.2	28.4	291	5.73	37.4	0.66	9.27

Table S3

Group	Time/d	PT/ s	APTT/s	TT/s	FIB/ g L^{-1}
Control	0	11.0±0.7	26.2±3.1	23.8±1.2	2.34±0.33
Low dose		11.3±0.7	27.0±1.9	24.0±1.2	2.13±0.17
Medium dose		10.9±0.5	27.7±2.6	23.2±1.0	2.66±0.62
High dose		11.4±0.6	28.2±3.3	24.5±2.0	2.30±0.57
Control	7	11.4±0.6	27.3±3.4	23.2±1.4	4.22±2.08
Low dose		11.9±0.6	27.5±2.3	24.3±0.7	3.66±1.32
Medium dose		11.3±0.7	29.2±2.7	24.8±0.4	4.29±1.26
High dose		11.8±0.8	27.7±4.0	23.7±1.1	3.85±0.29
Control	14	11.6±0.4	31.8±6.3	23.4±2.0	3.84±1.98
Low dose		11.7±0.5	30.6±2.1	22.7±1.2	3.59±1.52
Medium dose		11.3±0.5	32.4±3.9	23.5±1.8	3.35±0.63
High dose		11.5±0.5	31.3±4.3	22.6±1.4	3.17±0.52
Control	28	11.5±0.6	32.4±4.1	23.4±0.5	3.40±1.70
Low dose		11.7±0.7	32.3±3.1	24.0±1.3	3.17±1.08
Medium dose		11.3±0.5	33.4±2.7	23.5±1.1	2.87±0.66
High dose		11.7±0.5	33.6±3.0	24.2±2.6	2.50±0.38
Control	42	12.2	31.7	23.4	2.18
Low dose		12.2	30.9	25.1	2.06
Medium dose		12.0	31.4	23.7	2.42
High dose		12.6	31.8	22.8	2.05

Table S4

Group	Time/d	TBIL μmol/L	TP g/L	ALB g/L	GLOB g/L	A/G	ALP U/L	GGT U/L	UREA mmol/L
Control	0	1.8±0.3	68.6±1.7	39.1±1.7	29.5±2.7	1.3±0.2	380±135	49±18	6.90±1.3
Low dose		1.7±0.6	67.0±2.7	37.2±2.0	29.9±3.0	1.3±0.2	377±207	48±7	6.82±0.9
Medium		2.0±0.4	70.9±4.2	38.8±4.5	32.1±4.1	1.2±0.3	553±264	52±12	5.91±1.1
High dose		2.5±1.0	70.0±3.9	37.6±1.9	32.4±3.9	1.2±0.2	399±136	54±21	6.55±0.9
Control	7	2.1±0.7	64.4±7.9	32.4±8.5	32.0±2.5	1.0±0.3	390±76	39±15	6.66±1.0
Low dose		2.6±0.8	64.2±3.0	32.2±3.9	32.0±1.4	1.0±0.2	375±219	38±6	5.77±1.0
Medium		2.1±0.9	69.1±5.2	34.0±3.4	35.2±5.2	1.0±0.2	506±200	44±12	6.31±1.6
High dose		2.8±0.4	71.2±1.7	36.6±1.8	34.6±2.9	1.1±0.1	422±121	43±12	6.11±1.0
Control	14	1.9±1.0	70.8±3.1	32.9±8.3	37.9±7.7	0.9±0.4	463±102	40±18	6.07±1.1
Low dose		1.9±0.6	69.2±2.1	32.4±6.2	36.8±5.0	0.9±0.3	434±214	37±4	6.84±0.9
Medium		1.5±0.3	72.5±5.1	35.1±3.1	37.5±4.4	1.0±0.1	625±235	46±12	7.20±2.1
High dose		1.7±0.5	71.4±1.7	35.8±2.6	35.6±1.9	1.0±0.1	488±154	42±15	5.89±0.3
Control	28	2.2±1.2	69.9±4.3	33.2±9.0	36.7±7.8	1.0±0.4	455±54	43±17	6.86±1.4
Low dose		1.9±0.5	70.5±1.9	32.5±6.9	38.0±7.1	0.9±0.3	410±188	39±7	6.23±0.7
Medium		1.7±0.4	70.8±3.2	35.4±3.0	35.4±3.3	1.0±0.2	574±181	46±15	6.57±0.2
High dose		2.4±0.5	73.1±4.5	37.6±3.6	35.5±2.8	1.1±0.1	443±157	46±17	6.25±1.1
Control	42	2.1	71.4	40.1	31.3	1.3	327	68	5.94
Low dose		1.5	70.5	37.8	32.7	1.2	357	47	6.51
Medium		1.4	72.5	39.2	33.3	1.2	577	47	6.47
High dose		2.4	74.6	42.2	32.4	1.4	312	38	5.27

Table S5

Group	Time/d	CREA μmol/L	GLU mmol/L	TG mmol/L	CHOL mmol/L	K^+ mmol/L	Na^+ mmol/L	Cl^- mmol/L
Control	0	49.9±12.2	4.46±1.51	0.22±0.05	2.98±0.68	4.6±0.4	148±5	107±2
Low dose		53.5±6.5	4.63±0.72	0.29±0.22	2.88±0.47	4.7±0.4	147±2	107±2
Medium dose		53.6±6.8	4.28±0.92	0.31±0.19	3.28±0.62	4.6±0.8	147±4	106±1
High dose		50.6±8.4	4.52±1.12	0.30±0.10	3.23±1.01	4.9±0.6	146±3	106±1
Control	7	45.0±8.4	4.16±0.48	0.51±0.35	2.37±0.33	4.6±0.7	145±2	106±3
Low dose		49.4±7.0	4.53±0.99	0.24±0.19	2.38±0.33	4.9±0.5	146±2	107±1
Medium dose		48.0±5.6	4.08±0.61	0.42±0.28	2.78±0.38	4.9±0.7	145±4	106±2
High dose		48.6±8.2	4.69±0.77	0.26±0.10	2.90±0.96	5.0±0.4	146±3	106±2
Control	14	44.3±7.3	3.86±0.51	0.37±0.16	2.64±0.52	4.5±0.5	145±4	106±2
Low dose		49.4±6.7	3.83±0.99	0.27±0.18	2.36±0.49	4.5±0.5	146±3	107±2
Medium dose		52.6±6.0	3.81±0.66	0.40±0.15	2.77±0.38	4.6±0.6	148±4	108±2
High dose		48.1±8.8	3.76±0.68	0.38±0.16	2.65±0.75	4.7±0.4	142±2	104±1
Control	28	48.6±10.5	3.13±0.52	0.60±0.35	2.29±0.32	4.8±0.3	143±3	106±3
Low dose		53.6±9.1	3.32±0.59	0.28±0.10	2.31±0.30	5.0±0.5	143±2	106±2
Medium dose		52.4±6.5	3.50±0.48	0.49±0.21	2.61±0.24	5.0±0.6	143±3	106±1
High dose		54.9±9.2	3.52±1.22	0.40±0.10	2.92±0.77	4.8±0.3	144±2	105±2
Control	42	65.2	4.40	0.20	2.80	4.4	147	110
Low dose		66.8	4.78	0.21	2.07	4.2	147	108
Medium dose		67.9	4.65	0.31	2.28	4.1	146	109
High dose		68.2	4.31	0.20	3.49	4.1	146	107

Table S6

Group	Animal	Color	turbidity	pH	NIT	GLU mmol/L	SG	BLD mg/L	PRO g/L	BIL umol/L	URO umol/L	KET mmol/L	LEU Leu/uL
Control	1201	C	-	8.0	-	N	1.006	-	-	-	N	-	NEG.
	1202	Y	+1	9.0	+1	N	1.022	-	+-	-	N	-	NEG.
	1203	LY	+1	8.5	+1	N	1.007	-	-	-	N	-	NEG.
	1104	C	+1	8.5	-	N	1.009	-	-	-	N	-	NEG.
	1105	Y	+2	8.5	-	N	1.026	-	+-	-	N	-	NEG.
	1106	LY	+1	8.0	+1	N	1.006	-	-	-	N	-	NEG.
Low dose	2207	Y	+1	9.0	+1	N	1.025	-	+1	-	N	-	75
	2208	Y	+1	7.5	-	N	1.006	+3	-	-	N	-	75
	2209	LY	+1	8.5	+2	N	1.010	-	-	-	N	-	NEG.
	2110	LY	-	8.5	-	N	1.012	-	-	-	N	-	NEG.
	2111	LY	+1	8.0	-	N	1.009	+-	-	-	N	-	NEG.
	2112	C	+1	8.5	+1	N	1.006	-	-	-	N	-	NEG.

Table S7

Group	Animal	Color	turbidity	pH	NIT	GLU mmol/L	SG	BLD mg/L	PRO g/L	BIL umol/L	URO umol/L	KET mmol/L	LEU Leu/uL
Medium dose	3213	C	+1	8.0	-	N	1.008	-	-	-	N	-	500
	3214	C	+1	7.5	+1	N	1.004	-	-	-	N	-	NEG.
	3215	C	+1	8.0	-	N	1.007	-	-	-	N	-	250
	3116	LY	+1	9.0	+2	N	1.011	-	-	-	N	-	NEG.
	3117	LY	-	9.0	-	N	1.010	-	-	-	N	-	NEG.
	3118	Y	+1	8.5	-	N	1.022	-	-	-	N	-	NEG.
High dose	4219	LY	+2	7.5	+2	N	1.005	+1	-	-	N	-	NEG.
	4220	Y	+2	8.0	-	N	1.009	+1	-	-	N	-	250
	4221	C	-	8.5	-	N	1.006	-	-	-	N	-	NEG.
	4122	LY	+1	9.0	-	N	1.009	-	-	-	N	-	NEG.
	4123	LY	+1	8.0	-	N	1.007	-	-	-	N	-	NEG.
	4124	LY	-	8.5	-	N	1.010	-	-	-	N	-	NEG.
